# Endovascular thrombectomy in selected patients with active cancer and thrombocytopenia: outcomes under an institutional platelet transfusion practice

**DOI:** 10.3389/fneur.2026.1860736

**Published:** 2026-06-23

**Authors:** Hyung Jun Kim, Tae-Jin Song, Jin-Soo Lee, Keon-Ha Kim, Woo-Keun Seo, Pyoung Jeon, Gyeong-Moon Kim, Jong-Won Chung, Oh Young Bang

**Affiliations:** 1 Department of Neurology, Samsung Medical Center, School of Medicine, Sungkyunkwan University, Seoul, Republic of Korea; 2 Department of Neurology, Seoul Hospital, Ewha Womans University College of Medicine, Seoul, Republic of Korea; 3 Graduate Program in System Health Science and Engineering, Ewha Womans University, Seoul, Republic of Korea; 4 Department of Neurology, Ajou University College of Medicine, Suwon, Republic of Korea; 5 Department of Radiology, Samsung Medical Center, School of Medicine, Sungkyunkwan University, Seoul, Republic of Korea

**Keywords:** acute stroke management, cancer, endovascular thrombectomy, first-pass effect, thrombocytopenia

## Abstract

**Background and purpose:**

Thrombocytopenia is common in patients with active cancer and large vessel occlusion (LVO), raising safety concerns regarding endovascular thrombectomy (EVT). Therefore, we compared the procedural safety and clinical outcomes of EVT between patients with active cancer with and without thrombocytopenia.

**Methods:**

This retrospective multicenter study analyzed patients with active cancer who underwent EVT for anterior-circulation LVO between 2017 and 2024. Patients were categorized according to the presence of thrombocytopenia (<150 × 10^9^/L). All patients with moderate-to-severe thrombocytopenia (<100 × 10^9^/L, n = 14) received a transfusion of 6 units of platelet concentrates. Propensity score matching (PSM) was used to balance baseline characteristics. The primary endpoint was symptomatic intracranial hemorrhage (sICH). Secondary endpoints included any hemorrhagic transformation (HT) and 3-month clinical outcomes, including modified Rankin Scale (mRS) distribution, favorable outcome (mRS 0–2), and mortality.

**Results:**

Among 98 patients with active cancer undergoing EVT, PSM yielded 40 balanced pairs. Rates of hemorrhagic complications were numerically similar between the groups, with no statistically significant differences observed in HT (*p* > 0.99) or sICH (12.5% vs. 10%; *p* > 0.99). At 90 days, the thrombocytopenia group showed a worse functional status (median [mRS], 6 [interquartile range {IQR} 3–6] vs. 3 [IQR 1–6]; *p* = 0.029), with nonsignificant trends toward fewer favorable outcomes (mRS 0–2: 17.5% vs. 37.5%; *p* = 0.080) and higher mortality (55% vs. 37.5%; *p* = 0.179). However, adjusted Firth penalized logistic regression analyses using baseline characteristics as covariates showed that thrombocytopenia was not significantly associated with hemorrhagic complications. In an exploratory analysis of post-EVT clinical outcomes, FPE was associated with lower 3-month mortality (adjusted OR, 0.153; *p* = 0.008) and favorable outcome (adjusted OR, 6.353; *p* = 0.003).

**Conclusion:**

In this exploratory study of selected patients with active cancer undergoing EVT for anterior-circulation LVO, thrombocytopenia was not associated with a statistically demonstrable increase in hemorrhagic complications. However, these findings should be interpreted cautiously because of limited event numbers, residual confounding, and the absence of an untreated thrombocytopenic comparator group.

## Introduction

Patients with active cancer have an increased risk of acute ischemic stroke (AIS) compared with the general population (1, 2). This elevated risk is frequently associated with a cancer-related hypercoagulable state (2, 3). Consequently, patients with stroke and active malignancy often have a worse overall prognosis and higher mortality rates than those without cancer (3–6). Endovascular thrombectomy (EVT) is the established standard of care for acute AIS caused by large vessel occlusion (LVO) (5, 7). However, robust data regarding the safety and efficacy of EVT, specifically in patients with cancer, remain scarce because this population has been frequently excluded from major randomized controlled trials (5, 8, 9).

A critical and increasingly frequent clinical dilemma in this population is concomitant thrombocytopenia, which can result from chemotherapy, bone marrow infiltration, or disseminated intravascular coagulation (4, 10, 11). There is a prevailing concern that a low platelet count substantially increases the risk of hemorrhagic complications such as hemorrhagic transformation (HT) or symptomatic intracranial hemorrhage (sICH) during or immediately after EVT (12, 13). However, to the best of our knowledge, no studies to date have focused on the procedural safety and clinical outcomes of EVT in patients with active cancer presenting with thrombocytopenia (14). The number of clinical encounters in this specific subgroup is expected to increase significantly, driven by several converging factors. Advancements in oncological care have steadily expanded the population of individuals living with active cancer (5, 11). Simultaneously, the increasingly widespread use of diverse chemotherapy regimens has resulted in a higher prevalence of treatment-associated thrombocytopenia among those at risk of stroke (15). This epidemiological shift is further reinforced by the current clinical guidelines, which increasingly categorize these patients as EVT eligible, promoting an increasing clinical trend toward recanalization, even in this traditionally high-risk cohort (3, 7, 11). In clinical practice, low platelet count is often considered during EVT decision-making because of concerns regarding hemorrhagic complications, although evidence specific to patients with active cancer remains limited (7).

This multicenter study was designed to address this knowledge gap and was based on the hypothesis that EVT may be selectively feasible in patients with active cancer and thrombocytopenia, particularly in those with moderate-to-severe thrombocytopenia managed under a preprocedural institutional platelet transfusion practice. The aim of this study was to compare the procedural safety and 3-month clinical outcomes of EVT between patients with active cancer with thrombocytopenia and those without thrombocytopenia, using propensity score matching (PSM) to reduce potential confounding.

## Methods

### Study design and population

This was a retrospective, multicenter study of patients with AIS and active cancer who underwent EVT for LVO. Using prospectively established and maintained stroke registries from three tertiary centers between January 2017 and December 2024, we consecutively enrolled eligible patients who received EVT during the study period and retrospectively analyzed their data. The inclusion criteria were: (1) adult patients (aged ≥18 years); (2) diagnosis of active cancer at the time of stroke; (3) radiologically confirmed LVO in the anterior circulation; (4) treatment with EVT; and (5) a prestroke modified Rankin Scale (mRS) score of 0–2. After application of the inclusion criteria, patients with missing key baseline or outcome data were excluded. Active cancer was defined as a diagnosis of cancer within 6 months prior to enrollment, recurrent or metastatic cancer, or current treatment with chemotherapy or radiotherapy. The study protocol was approved by the Institutional Review Board (IRB) of the lead center (Samsung Medical Center; IRB No. SMC 2016–02-104), and each participating center obtained approval from its respective local IRB.

### Data collection and definition

We collected demographic characteristics, vascular risk factors, baseline stroke severity, stroke etiology, time from last known normal to groin puncture, laboratory findings, cancer-related variables, and procedural variables. Cancer-related variables included primary cancer site, histological type, systemic metastasis, recent chemotherapy within 4 weeks, and evidence of cancer-associated hypercoagulability. EVT-related data were extracted from procedural reports and angiographic records, including the first-line thrombectomy strategy, use of direct aspiration, stent retriever, or combined technique, number of passes, final mTICI grade, successful recanalization, and first-pass effect. The primary variable was the presence of thrombocytopenia on admission for LVO, defined as a platelet count <150 × 10^9^/L (16). To evaluate dose–response relationships, patients were stratified by platelet count severity: mild (100–150 × 10^9^/L), moderate (50–100 × 10^9^/L), and severe (<50 × 10^9^/L) or by tertiles for specific association analyses. At the participating centers, patients with moderate-to-severe thrombocytopenia (<100 × 10^9^/L) were managed with a standardized institutional practice involving the transfusion of 6 units of platelet concentrates starting immediately before the EVT procedure (17). For all patients who underwent platelet transfusion, platelet concentrates were administered concurrently with the EVT procedure; groin puncture and thrombectomy were performed without waiting for confirmation of a post-transfusion platelet count. Platelet counts were reassessed after the procedure, and an additional 6 units of platelet concentrates were transfused if the platelet count remained below 100 × 10^9^/L. Preprocedural platelet transfusion for platelet counts <100 × 10^9^/L reflected institutional practice at the participating centers rather than a universally established EVT-specific guideline. Accordingly, the present study should be interpreted as evaluating EVT in thrombocytopenic patients managed under an institutional transfusion practice rather than untreated thrombocytopenia per se.

### Procedural and clinical outcomes

The primary safety endpoint was sICH. Secondary safety endpoints included any HT, classified into hemorrhagic infarction (HI) 1, HI 2, parenchymal hemorrhage (PH) 1, PH 2, and remote ICH. Secondary clinical endpoints included 3-month mRS distribution, favorable outcome (mRS 0–2), and mortality. Successful recanalization (mTICI 2b–3) and first-pass effect (FPE) were analyzed as secondary procedural outcomes. FPE was defined as successful recanalization (mTICI 2b–3) after a single pass of the EVT device without rescue therapy or additional passes (18). Safety outcomes included the incidences of HT and sICH confirmed by follow-up imaging. HT was classified according to the ECASS/Heidelberg criteria as HI 1 or 2, PH 1 or 2, or remote ICH (19). sICH was defined as any ICH, including remote ICH, associated with neurological deterioration, defined as an increase of ≥4 points in the NIHSS score, or death judged to be related to the hemorrhagic event (20).

### Statistical analysis

PSM was performed to reduce selection bias and achieve balanced baseline characteristics between the thrombocytopenia and non-thrombocytopenia groups. A 1:1 matching ratio was applied with a caliper width of 0.2 of the standard deviation of the logit of the propensity score. Variables included in the matching model were age, sex, initial NIHSS score, systemic metastasis, cancer histology, and recent chemotherapy, with particular attention paid to achieving balance in cancer-related status variables between the two groups. This resulted in 40 matched pairs (n = 80) for the primary analysis. Baseline balance before and after propensity score matching was assessed using standardized mean differences. Statistical testing was not used as the primary measure of baseline balance because *p* values are sample-size dependent and do not directly quantify covariate imbalance. Continuous variables were compared using the Student’s t-test or Mann–Whitney U test, and categorical variables were analyzed using the chi-square test or Fisher’s exact test, as appropriate. Univariable logistic regression analyses were first performed to assess associations between candidate variables and study outcomes. Given the limited number of outcome events, particularly for sICH and favorable outcome, multivariable analyses were performed using Firth penalized logistic regression to reduce small-sample bias and account for sparse-data issues. Given the limited number of outcome events, the events-per-variable ratio was low in some adjusted models, potentially resulting in unstable estimates. Although Firth penalized logistic regression was used to mitigate small-sample bias, the results of multivariable analyses should be interpreted with caution given these constraints. Adjusted models were constructed for HT, sICH, 3-month mortality, and favorable outcome. The primary adjusted models were constructed for the main safety and clinical outcomes using clinically relevant variables and variables with *p* < 0.10 in univariable analyses. Additional models including procedural efficacy variables were considered exploratory because these variables occur during EVT and may lie on the causal pathway. Although Firth penalized logistic regression was used to reduce small-sample bias, the results should be interpreted cautiously because of sparse outcome events and limited events per variable.

All statistical analyses were performed using R version 4.5.1, and a two-sided *p*-value <0.05 was considered statistically significant.

## Results

### Baseline characteristics and matching

**Figure 1:**
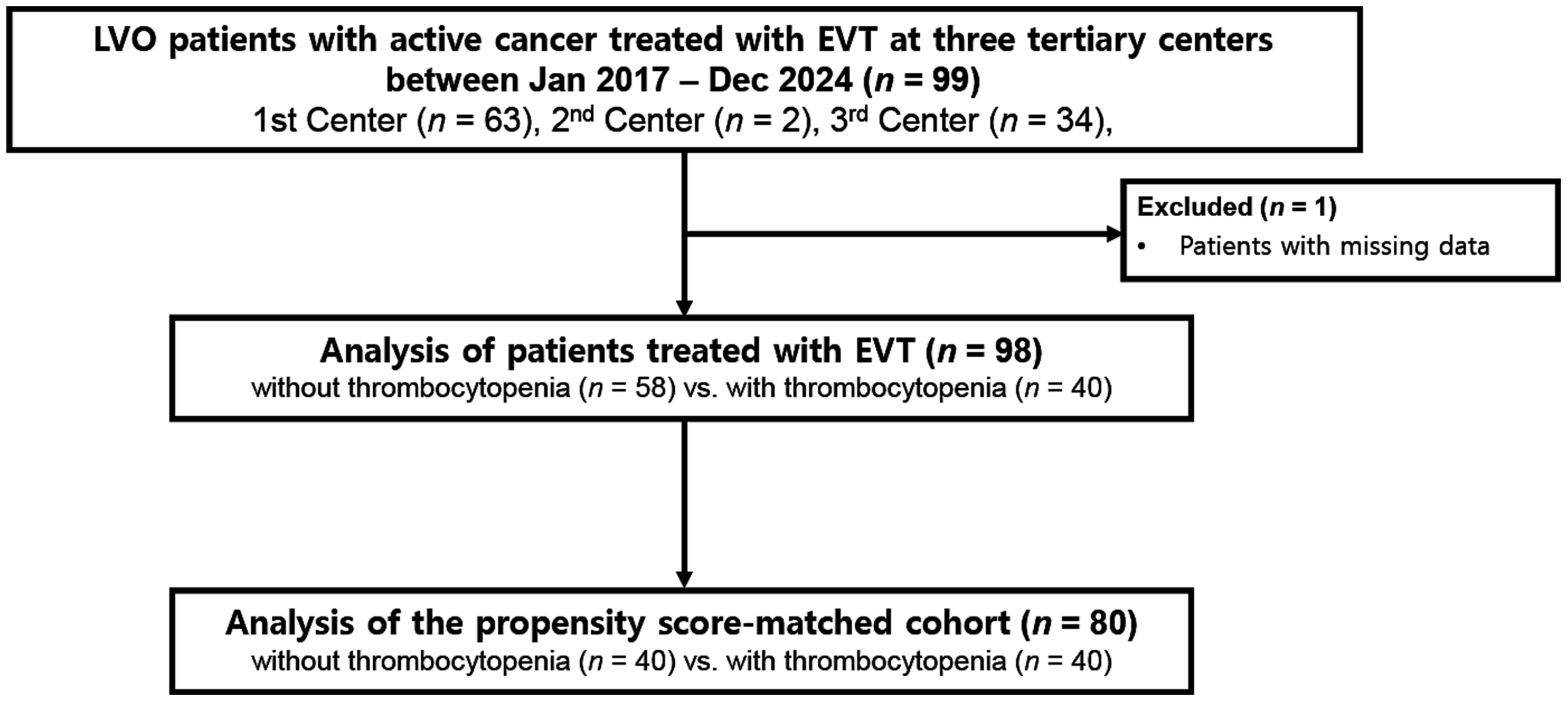
Patient flow chart. LVO, large vessel occlusion; EVT, endovascular thrombectomy.

Of the 99 initially identified patients, 98 were included in the final analysis after excluding one patient with missing data. A total of 98 patients with active cancer who underwent EVT for LVO were included. In accordance with the study eligibility criteria, all included patients had a prestroke modified Rankin Scale (mRS) score of 0–2. The median prestroke mRS score was 0 (interquartile range, 0–1) in the overall cohort. Among them, 40 patients (40.8%) presented with thrombocytopenia at the time of the procedure (Figure 1). Before PSM, the thrombocytopenia group tended to be younger (69.6 vs. 71.0 years) and had higher proportions of adenocarcinoma (52.5% vs. 34.5%) and chemotherapy within 4 weeks (85% vs. 67.2%) than the non-thrombocytopenia group. To control for these baseline differences, PSM yielded 40 pairs (n = 80). After PSM, most baseline characteristics, including age, stroke etiology, and primary cancer lesions, showed standardized mean differences (SMD) < 0.1, indicating improved balance. However, notable residual imbalance persisted in systemic metastasis (50% vs. 57.5%; SMD = 0.149) and hepatobiliary cancer (10% vs. 0%; SMD = 0.140). Given that systemic metastasis was subsequently identified as an independent predictor of 3-month mortality (adjusted OR, 5.501), this residual confounding should be considered when interpreting outcome differences between groups (Table 1).

**Table 1:** Baseline characteristics of patients with active cancer and large vessel occlusion, categorized by thrombocytopenia status, before and after propensity score matching.

Characteristic	Total (*N* = 98)	Before PSM		*SMD*	After PSM		*SMD*
		Without thrombocytopenia (*n* = 58)	With thrombocytopenia (*n* = 40)		Without thrombocytopenia (*n* = 40)	With thrombocytopenia (*n* = 40)	
Age, years	70.36 ± 10.89	71 ± 10.4	69.62 ± 11.7	0.124	70.08 ± 9.95	69.62 ± 11.72	0.041
Male sex	52 (52.5%)	30 (51.7%)	22 (55%)	0.065	19 (47.5%)	22 (55%)	0.095
Prestroke mRS	0 (0, 1)	0 (0, 1)	0 (0, 1)	0.012	0 (0, 1)	0 (0, 1)	0.008
Initial NIHSS score (IQR)	13 (8, 18)	13.5 (8.5, 18)	12 (8, 19)	0.065	14 (10, 18)	12 (8, 19)	0.011
Stroke etiology				0.130			0.035
Cryptogenic embolism	50 (51.0%)	27 (46.6%)	23 (57.5%)		21 (52.5%)	23 (57.5%)	
Cardio-embolism	39 (39.8%)	24 (41.4%)	15 (37.5%)		15 (37.5%)	15 (37.5%)	
Large artery atherosclerosis	9 (9.2%)	7 (12.07%)	2 (5%)		4 (10%)	2 (5%)	
Primary cancer lesion				0.205			0.140
Lung	20 (20.4%)	10 (17.2%)	10 (25%)		10 (25%)	10 (25%)	
Gastrointestinal	26 (26.5%)	14 (24.1%)	12 (30%)		7 (17.5%)	12 (30%)	
Hepatobiliary	8 (8.2%)	8 (13.8%)	0 (0%)		4 (10%)	0 (0%)	
Breast-gynecology	24 (24.5%)	12 (20.7%)	12 (30%)		11 (27.5%)	12 (30%)	
Urinary tract	14 (14.3%)	10 (17.2%)	4 (10%)		5 (12.5%)	4 (10%)	
Other	6 (6.1%)	4 (6.9%)	2 (5%)		3 (7.5%)	2 (5%)	
Cancer type							
Adenocarcinoma	41 (41.8%)	20 (34.5%)	21 (52.5%)	0.366	20 (50%)	21 (52.5%)	0.049
Systemic metastasis	46 (46.9%)	23 (39.7%)	23 (57.5%)	0.359	20 (50%)	23 (57.5%)	0.149
Chemotherapy within 4 weeks	73 (74.5%)	39 (67.2%)	34 (85%)	0.422	35 (87.5%)	34 (85%)	0.072
Procedural factor							
LNT to puncture	458.48 ± 457.03	472.36 ± 497.37	438.35 ± 396.57	0.076	439.57 ± 405.66	438.35 ± 396.57	0.003

EVT, endovascular thrombectomy; PSM, propensity score matching; SMD, standard mean difference; IQR, interquartile range; LNT, last normal time; NIHSS, National Institutes of Health Stroke Scale.

### Laboratory and procedural findings

In the thrombocytopenia group, the majority of cases were mild (65%), followed by moderate (27.5%) and severe (7.5%). The distribution of first-line thrombectomy strategy did not differ significantly between the groups (*p* = 0.155). Direct aspiration was more frequently applied in the thrombocytopenia group than in the non-thrombocytopenia group (52.5% vs. 35.0%), whereas stent retrievers were less commonly used (17.5% vs. 35.0%). The combined technique was used in 30.0% of the patients in both groups. Regarding procedural outcomes, the thrombocytopenia group had lower rates of successful reperfusion (mTICI 2b–3; 65.0% vs. 95.0%, *p* = 0.002) and FPE (17.5% vs. 50.0%, *p* = 0.005) (Supplemental Data 1). Among the 14 patients who received platelet transfusion, no transfusion-related complications were documented.

### Safety outcomes: hemorrhagic complications

sICH was observed in 12.5% of the thrombocytopenia group and 10% of the non-thrombocytopenia group, with no statistically significant difference between groups (*p* > 0.99) (Table 2). HT occurred in 35% (14/40) of patients in both groups (*p* > 0.99). The distributions of HT subtypes (HI1, HI2, PH1, PH2, and rICH) were also comparable between groups (Supplemental Data 2). Although hemorrhagic complication rates were numerically similar between groups, the study was not powered to exclude clinically meaningful differences, particularly for sICH. In adjusted Firth penalized logistic regression analyses, initial thrombocytopenia was not significantly associated with HT (adjusted odds ratio [OR], 1.069; 95% confidence interval [CI], 0.402–2.845; *p* = 0.894) or sICH (adjusted OR, 1.896; 95% CI, 0.408–8.809; *p* = 0.414) (Table 2). Furthermore, in the analysis stratifying patients with thrombocytopenia by platelet count tertiles, no significant associations were observed between thrombocytopenia severity and the incidence of HT or sICH (all *p* > 0.05) (Supplemental Data 3).

**Table 2:** Comparison of hemorrhagic complications and 3-month clinical outcomes in the propensity score-matched cohort.

Outcome	Without thrombocytopenia (*n* = 40)	With thrombocytopenia (*n* = 40)	*p*	Adjusted OR	*p*
Hemorrhagic complications					
HT	14 (35%)	14 (35%)	>0.99	1.069 (0.402–2.845)	0.894
sICH	4 (10%)	5 (12.5%)	>0.99	1.896 (0.408–8.809)	0.414
Clinical outcomes					
Median mRS score at 3 months (IQR)	3 (1, 6)	6 (3, 6)	0.029		
Favorable outcome (mRS score 0–2)	15 (37.5%)	7 (17.5%)	0.080	0.519 (0.148–1.818)	0.305
Mortality at 3 months	15 (37.5%)	22 (55%)	0.179	1.318 (0.431–4.026)	0.628
- Death in hospital	- 6 (40%)	- 7 (31.82%)			
- Death post-discharge	- 9 (60%)	- 15 (68.18%)			

OR, odds ratio; mTICI, modified Thrombolysis In Cerebral Infarction; HT, hemorrhagic transformation; sICH, symptomatic intracranial hemorrhage; mRS, modified Rankin scale; IQR, interquartile range.

The covariates included in the adjusted Firth penalized logistic regression model for hemorrhagic transformation were thrombocytopenia, and first-line technique.

The covariates included in the adjusted Firth penalized logistic regression model for sICH were thrombocytopenia, HbA1c, and LDL.

The covariates included in the adjusted Firth penalized logistic regression model for favorable outcomes were thrombocytopenia, initial NIHSS score, hemoglobin level, D-dimer level, and systemic metastasis.

The covariates included in the adjusted Firth penalized logistic regression model for mortality were thrombocytopenia, hemoglobin level, D-dimer level, systemic metastasis, and diabetes.

**Figure 2:**
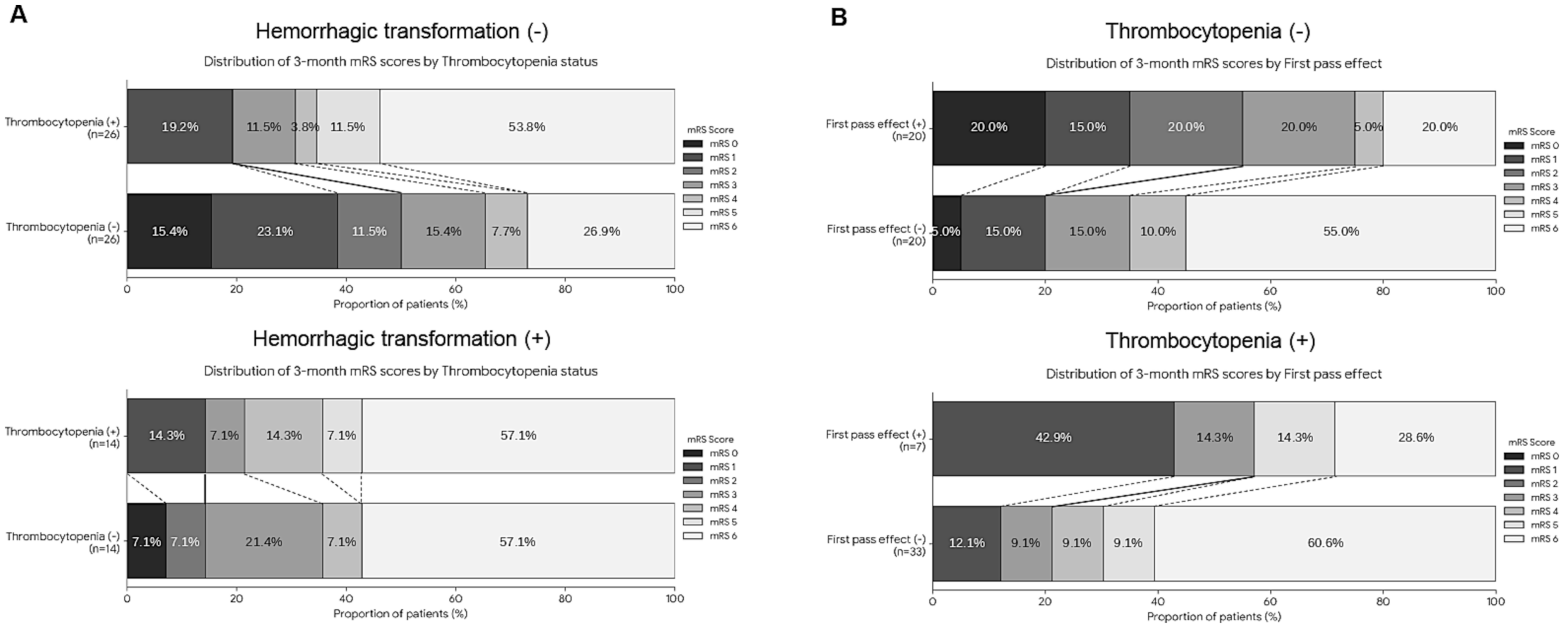
Distribution of 3-month favorable clinical outcome (modified Rankin Scale [mRS], 0–6) according to hemorrhagic transformation (HT) and first-pass effect (FPE) in the propensity score-matched cohort. **(A)** Stacked horizontal bars show the distribution of 3-month mRS scores by thrombocytopenia status, stratified by the presence of HT (HT - vs. HT +). **(B)** Within each platelet-status (thrombocytopenia - and thrombocytopenia +), stacked horizontal bars show the distribution of 3-month mRS scores by FPE (FPE + vs. FPE -).

The effect of HT on clinical outcomes was also analyzed (Figure 2A). In the absence of HT, the thrombocytopenia group still exhibited a higher mortality rate compared with the non-thrombocytopenia group (53.8% vs. 26.9%, respectively). When HT was present, both groups showed high 3-month mortality (57.1% for both), indicating that, while HT significantly compromised prognosis in both cohorts, the thrombocytopenia group maintained a higher baseline risk of poor outcomes due to systemic factors, independent of hemorrhagic complication occurrence.

### Clinical outcomes at 3 months: mortality and favorable outcome

The thrombocytopenia group exhibited a shift toward poorer clinical outcomes at 3-month (Supplemental Data 4). Specifically, the median mRS score was significantly higher in the thrombocytopenia group than in the non-thrombocytopenia group (6 [interquartile range {IQR}, 3–6] vs. 3 [IQR, 1–6], *p* = 0.029). Furthermore, although not statistically significant, favorable outcomes (mRS 0–2) were less frequent (17.5% vs. 37.5%, *p* = 0.080), and 3-month mortality was higher (55% vs. 37.5%, *p* = 0.179), in the thrombocytopenia group (Table 2).

In contrast to the univariate findings, adjusted Firth penalized logistic regression analysis revealed that initial thrombocytopenia was not independently associated with 3-month mortality (adjusted OR, 1.318; 95% CI, 0.431–4.026; *p* = 0.628) or favorable outcome (adjusted OR, 0.519; 95% CI, 0.148–1.818; *p* = 0.305) (Table 2). Similarly, in the analysis stratified by platelet count tertiles, the severity of thrombocytopenia showed no dose–response relationship or significant association with either 3-month mortality or favorable outcome in the adjusted models (all *p* > 0.05) (Supplemental Data 3). In exploratory adjusted Firth penalized logistic regression analyses including clinically relevant covariates (Table 3), first-line thrombectomy technique was not significantly associated with 3-month mortality or favorable outcome. In contrast, FPE was significantly associated with lower 3-month mortality (adjusted OR, 0.153; 95% CI, 0.038–0.614; *p* = 0.008) and higher odds of favorable outcome (adjusted OR, 6.353; 95% CI, 1.867–21.621; *p* = 0.003). Furthermore, systemic metastasis was associated with 3-month mortality (adjusted OR, 5.501; 95% CI, 1.787–16.940; p = 0.003). Regarding functional recovery, lower initial NIHSS scores (*p* = 0.034) and D-dimer levels (*p* = 0.046) were independently associated with favorable outcome.

**Table 3:** Exploratory adjusted Firth penalized logistic regression analyses of factors associated with 3-month clinical outcomes after endovascular thrombectomy.

Variable	HT		sICH		3-month mortality		3-month favorable outcome	
	Adjusted OR	*p*	Adjusted OR	*p*	Adjusted OR	*p*	Adjusted OR	*p*
Initial thrombocytopenia	**0.719 (0.247–2.098)**	**0.546**	**1.896 (0.408–8.809)**	**0.414**	**1.021 (0.323–3.224)**	**0.972**	**0.677 (0.175–2.617)**	**0.571**
Initial NIHSS score	1.012 (0.945–1.084)	0.735	0.978 (0.870–1.099)	0.707	1.019 (0.941–1.104)	0.646	**0.898 (0.814–0.992)**	**0.034**
IV thrombolysis	1.736 (0.389–7.754)	0.470	**5.586 (1.199–16.666)**	**0.003**	1.160 (0.216–6.235)	0.863	0.316 (0.030–3.351)	0.339
Successful recanalization	1.276 (0.361–4.506)	0.705	0.265 (0.046–1.501)	0.134	3.026 (0.546–16.778)	0.205	0.675 (0.071–6.455)	0.733
First-pass effect	**0.264 (0.079–0.883)**	**0.031**	0.433 (0.075–2.492)	0.349	**0.153 (0.038–0.614)**	**0.008**	**6.353 (1.867–21.621)**	**0.003**
LNT to puncture	1.001 (1.000–1.002)	0.173	1.001 (1.000–1.003)	0.116	1.000 (0.998–1.001)	0.691	0.999 (0.997–1.001)	0.350
First-line technique								
- Stent retriever	Reference		Reference		Reference		Reference	
- Direct aspiration	0.843 (0.261–2.721)	0.775	0.386 (0.066–2.272)	0.293	0.512 (0.135–1.943)	0.325	0.807 (0.306–7.219)	0.623
- Combined technique	**0.242 (0.058–1.008)**	**0.051**	0.245 (0.032–1.874)	0.175	1.420 (0.338–5.965)	0.632	0.888 (0.251–8.131)	0.688
Adenocarcinoma	1.154 (0.460–2.896)	0.761	0.601 (0.121–2.991)	0.534	0.504 (0.165–1.538)	0.228	0.688 (0.202–2.348)	0.551
Systemic metastasis	1.235 (0.489–3.114)	0.655	1.304 (0.267–6.358)	0.743	**5.501 (1.787–16.940)**	**0.003**	0.908 (0.227–3.642)	0.892
Initial D-dimer	1.003 (0.973–1.034)	0.834	1.038 (0.997–1.081)	0.067	1.036 (0.993–1.081)	0.105	**0.925 (0.856–0.999)**	**0.046**
HbA1c*	1.182 (0.800–1.748)	0.401	**1.019 (1.001–1.038)**	**0.043**	1.548 (0.847–2.828)	0.156	0.548 (0.232–1.292)	0.169

HT, hemorrhagic transformation; sICH, symptomatic intracranial hemorrhage; OR, odds ratio; NIHSS, National Institutes of Health Stroke Scale; IV, intravenous; LNT, last normal time; HbA1c, hemoglobin A1c.

The covariates included in the adjusted Firth penalized logistic regression model for hemorrhagic transformation were thrombocytopenia, first-line technique, and first-pass effect.

The covariates included in the adjusted Firth penalized logistic regression model for sICH were thrombocytopenia, HbA1c, and LDL.

The covariates included in the adjusted Firth penalized logistic regression model for mortality were thrombocytopenia, hemoglobin level, D-dimer level, systemic metastasis, diabetes, and first-pass effect.

The covariates included in the adjusted Firth penalized logistic regression model for favorable outcome were thrombocytopenia, initial NIHSS score, hemoglobin level, D-dimer level, systemic metastasis, and first-pass effect.

*HbA1c was entered as a continuous variable per 0.1% increase.

Bold values indicate statistical significance (*p* < 0.05).

Detailed analysis of clinical outcome distributions revealed that FPE was a critical determinant of 3-month mRS scores, regardless of platelet status (Figure 2B). In the non-thrombocytopenia group, FPE (+) was associated with a higher proportion of favorable outcomes (mRS 0–2) compared with FPE (−) (55.0% vs. 20.0%). This trend was even more pronounced in the thrombocytopenia group; while FPE (−) was associated with a 60.6% mortality rate (mRS 6), those achieving FPE showed a marked improvement in functional distribution, with 42.9% achieving an mRS of 1.

### Exploratory subgroup analysis by thrombocytopenia etiology

Exploratory subgroup analyses were performed using the thrombocytopenia cohort (n = 40) according to suspected thrombocytopenia etiologies, including systemic metastasis, recent chemotherapy (within 4 weeks), and evidence of hypercoagulability (Supplemental Data 5). Hemorrhagic complications did not differ significantly across these strata, with comparable rates of HT and sICH (all *p* > 0.05). In contrast, clinical outcomes varied by etiology; patients with systemic metastasis had significantly worse 3-month clinical outcomes (median mRS 6 [IQR 5–6] vs. 4 [IQR 1–6], *p* = 0.028) and higher 3-month mortality (69.6% vs. 35.3%, *p* = 0.067) than those without metastasis. Similarly, patients with hypercoagulability showed poorer 3-month clinical outcomes (median mRS, 6 [IQR, 5–6] vs. 4 [IQR, 1–6], *p* = 0.046) and a lower proportion of favorable outcomes (8.0% vs. 33.3%, *p* = 0.107) than those without hypercoagulability. In this exploratory analysis, recent chemotherapy within 4 weeks was not significantly associated with hemorrhagic complications or 3-month clinical outcomes.

## Discussion

Our findings suggest that EVT may be feasible in selected patients with active cancer and thrombocytopenia when managed under a preprocedural institutional platelet transfusion practice, rather than establishing the safety of thrombocytopenia itself in an unselected EVT population. These findings support the feasibility of EVT for this vulnerable population under such controlled conditions. Importantly, achieving FPE was associated with a favorable prognosis in this high-risk cohort.

The comparable bleeding risk between the thrombocytopenia and non-thrombocytopenia groups has an important clinical implication. Although pre-procedural platelet transfusion in selected severe cases may have contributed to the low hemorrhagic event rate, our findings suggest that thrombocytopenia, when managed under a preprocedural institutional platelet transfusion practice in selected EVT-treated patients, may not necessarily preclude EVT. This observation may reflect the mechanistic distinction between intravenous thrombolysis (IVT) and EVT. While IVT increases bleeding risk through systemic coagulopathy and is not recommended at platelet counts ≤100,000/μL (7), post-EVT hemorrhage is more strongly related to local procedural factors such as vessel injury and reperfusion damage than to baseline systemic coagulation status (21). Similarly, previous studies have reported that platelet count is not an independent predictor of sICH after EVT (22–24). The relatively high sICH rates observed in both groups may reflect the particularly high-risk nature of this cohort, which consisted exclusively of patients with active cancer undergoing EVT and included a substantial burden of systemic disease and cancer-related coagulopathy. This safety profile aligns with advances in endovascular technology, from earlier, more traumatic devices (the Merci Retriever) (25, 26) to modern stent retrievers, aspiration catheters, and combined techniques, which aim to reduce endothelial traction and unnecessary passes (27–30). In our cohort, direct aspiration was numerically more frequently used as the first-line thrombectomy technique in the thrombocytopenia group than in the non-thrombocytopenia group, whereas stent retriever use was less frequent; however, the overall distribution of first-line thrombectomy techniques did not differ significantly between groups. In the multivariable analysis, the combined technique was associated with numerically lower odds of hemorrhagic transformation compared with stent retriever use, although this did not reach statistical significance. These observations may reflect an operator preference for techniques perceived to reduce endothelial traction and procedural manipulation in patients considered to be at higher bleeding risk; however, this interpretation remains speculative given the retrospective design.

Another technological advancement in EVT is enhancement of the FPE; the achievement of successful recanalization in a single pass (18). Previous studies of EVT in patients with active cancer or cancer-related stroke have suggested that both procedural success and treatment efficiency are important determinants of outcome (31, 32). In the present study, FPE was more strongly associated with 3-month mortality and favorable functional outcome than successful recanalization alone. This finding may reflect the particular importance of rapid and efficient reperfusion in patients with active cancer, who may have limited physiological reserve, cancer-associated coagulopathy, and poor systemic prognosis. For high-risk patients, including those with thrombocytopenia, minimizing the number of procedural passes may help reduce iatrogenic vessel injury and facilitate timely reperfusion (33). Consequently, strategies aimed at maximizing FPE, such as the utilization of balloon guide catheters (34) or optimized multi-device combinations, should be actively prioritized to improve clinical outcomes in patients with cancer undergoing EVT (29, 35).

Recent binational registry data similarly showed that patients with cancer undergoing EVT had comparable reperfusion and symptomatic intracranial hemorrhage rates to those without cancer, but worse functional outcomes and higher mortality, suggesting that systemic cancer-related factors may substantially influence prognosis after technically successful EVT. Notably, that study also found that higher platelet count was associated with favorable outcomes among patients with cancer, supporting our interpretation that thrombocytopenia may reflect systemic disease burden or cancer-associated coagulopathy rather than bleeding vulnerability alone (36). Although thrombocytopenia was not independently associated with hemorrhagic complications or 3-month outcomes in the adjusted Firth penalized logistic regression analyses, unadjusted analyses showed a consistent trend toward poorer outcomes (Supplemental Data 4). Our findings should also be interpreted in the context of prior literature reporting higher symptomatic hemorrhage rates in thrombocytopenic patients undergoing thrombectomy. Differences in patient selection, the exclusive inclusion of active cancer, and the routine use of preprocedural platelet transfusion in moderate-to-severe thrombocytopenia may partly account for these discrepant observations. This aligns with findings from previous studies on thrombocytopenia in the general population (21, 22, 37), and supports the interpretation that thrombocytopenia may function primarily as a surrogate marker of advanced systemic illness (3, 9, 12, 14). Thrombocytopenia in patients with cancer may often be a consequence of consumptive coagulopathy associated with an underlying potential hypercoagulable state, which may involve platelet activation and consumption (1, 4, 38). In patients with cancer-associated stroke, thrombocytopenia may reflect high disease burden and coagulopathy/hypercoagulability, which could contribute to more resistant thrombus characteristics and lower procedural efficiency (39–41). Although this observation should be interpreted cautiously because procedural variables were not included in the PSM and may reflect post-baseline treatment-related factors, it suggests that thrombocytopenia in active cancer may represent more than a bleeding-risk marker.

The marked differences in recanalization and FPE despite baseline matching suggest that thrombocytopenia may be acting as a marker of systemic cancer burden, hypercoagulability, or altered thrombus composition rather than bleeding vulnerability alone. Consistent with this concept, the thrombocytopenia group in our study had a substantially lower FPE and required more passes than the non-thrombocytopenia group. In fact, histopathological analyses of thrombi retrieved from stroke patients with active cancer, particularly those categorized as cancer-related cryptogenic stroke or non-bacterial thrombotic endocarditis (NBTE), frequently reveal a high proportion of fibrin and platelets (39–41). Therefore, the observed reduction in platelet count may not directly correspond to an increased risk of clinical bleeding, but rather reflect ongoing thrombotic activity. In line with this mechanism, the thrombi formed under such conditions are likely to be firmer and associated with greater frictional resistance and adhesion to the vessel wall. This hypothesis is supported by our procedural data, which showed a significantly lower first-pass effect (17.5% vs. 50%) and a higher number of passes required in the thrombocytopenia group (Supplemental Data 1). These findings further support the notion that systemic cancer burden and coagulopathy, rather than thrombocytopenia, may largely determine prognosis after EVT.

This study has some limitations. First, it was a retrospective, multicenter study, which carries an inherent risk of selection bias and unmeasured confounding factors, despite our efforts to minimize these issues through PSM. Residual confounding remained likely, particularly because substantial between-group differences persisted in procedural efficacy variables after matching. Furthermore, despite PSM, residual imbalance remained in key cancer-related variables, notably systemic metastasis (SMD = 0.149), which was identified as an independent predictor of 3-month mortality. Although we adjusted for systemic metastasis in the multivariable models, the combined effect of residual confounding in the matched cohort and limited sample size may have influenced the observed between-group differences in clinical outcomes. Second, although we included patients from three tertiary centers, the sample size, particularly that of the moderate-to-severe thrombocytopenia subgroup, was relatively small (n = 14). Consequently, because only 9 sICH events occurred in the PSM cohort, the statistical power to detect minor to moderate differences in hemorrhagic risk was insufficient. Therefore, the absence of a statistically significant difference should not be interpreted as definitive clinical equivalence, and the possibility of a type II error remains. Third, although this was a multicenter study, most patients were enrolled from two high-volume centers, and one participating center contributed only a small number of patients. Fourth, the heterogeneity of histological types, cancer types and stages included in the study may have influenced the findings. While we adjusted for major cancer-related variables, the specific pathophysiological mechanisms of thrombosis (e.g., mucin production in adenocarcinomas vs. others) could not be individually analyzed owing to the limited sample size. Fifth, because all patients with moderate-to-severe thrombocytopenia received platelet transfusion according to institutional practice, the present study cannot separate the effect of thrombocytopenia from that of platelet transfusion. Moreover, there was no comparator group of thrombocytopenic patients who underwent EVT without transfusion. Therefore, our findings should be interpreted as outcomes among selected thrombocytopenic patients treated under an institutional transfusion practice, not as evidence that the institutional transfusion practice itself improves safety or efficacy. Finally, decision making regarding EVT performance might have varied among the participating centers and physicians, potentially influencing the inclusion of patients with poor systemic conditions. Because the study included only patients who ultimately underwent EVT, the cohort likely underrepresents patients with severe thrombocytopenia or poor systemic status who were not selected for EVT, potentially biasing the observed safety profile toward a more favorable estimate. However, across all three institutions, thrombocytopenia alone was not a determining factor in the decision to withhold EVT; rather, a consistent institutional platelet transfusion practice was followed for cases of moderate-to-severe thrombocytopenia prior to the procedure.

## Conclusion

In this selected cohort of patients with active cancer who underwent EVT, thrombocytopenia managed under a preprocedural institutional platelet transfusion practice was not associated with a statistically demonstrable increase in hemorrhagic complications or worse adjusted 3-month outcomes. Furthermore, it is essential to adopt clinical strategies that prioritize achieving a FPE to optimize both procedural safety and clinical outcomes in this vulnerable population. However, these findings should be interpreted cautiously because of limited event numbers, potential selection bias, residual confounding, and the inability to separate the effects of thrombocytopenia from those of platelet transfusion. Treatment decisions should therefore remain individualized.

## Data Availability

The raw data supporting the conclusions of this article will be made available by the authors, without undue reservation.
