# Supplementary material for: Endovascular thrombectomy in selected patients with active cancer and thrombocytopenia: outcomes under an institutional platelet transfusion practice
Source: Front Neurol. 2026 Jun 23;17:1860736. doi: 10.3389/fneur.2026.1860736 (PMC13337439; doi:10.3389/fneur.2026.1860736)
Supplement: Supplementary file 1 [file Table_1.docx]

**Endovascular Thrombectomy in Selected Patients With Active Cancer and Thrombocytopenia: Outcomes Under an Institutional Platelet Transfusion Practice**

**Supplemental DATA**

**Supplemental Data 1**. Comparison of comorbidities, laboratory profiles, and procedural efficacy in the matched cohort.

|  | Without thrombocytopenia  (N=40) | With thrombocytopenia  (N=40) | *p* |
| --- | --- | --- | --- |
| Risk factor |  |  |  |
| Hypertension | 24 (60%) | 19 (47.5%) | 0.370 |
| Diabetes | 6 (15%) | 9 (22.5%) | 0.567 |
| Hyperlipidemia | 16 (40%) | 8 (20%) | 0.088 |
| Atrial fibrillation | 6 (15%) | 9 (22.5%) | 0.567 |
| Systemic thromboembolism |  |  |  |
| DVT | 4 (10%) | 2 (5%) | 0.671 |
| PTE | 2 (5%) | 3 (7.5%) | 0.999 |
| Laboratory results |  |  |  |
| Hb | 11.25 ± 2 | 10.79 ± 1.98 | 0.297 |
| D-dimer, ug/mL | 7.29 ± 11.12 | 17.27 ± 17.06 | 0.003 |
| HbA1c | 5.85 ± 0.69 | 6.28 ± 1.53 | 0.115 |
| LDL | 86.6 ± 33.77 | 80.8 ± 41.01 | 0.492 |
| hsCRP | 4.37 ± 6.19 | 5.26 ± 7.89 | 0.575 |
| Platelet count | 254.03 ± 86.15 | 107.42 ± 34.88 | <0.001 |
| Mild thrombocytopenia (100 ~ 150× 10^9^/L) | NA | 26 (65%) |  |
| Moderate thrombocytopenia (50 ~ 100 × 10^9^/L) | NA | 11 (27.5%) |  |
| Severe thrombocytopenia (< 50 × 10^9^/L) | NA | 3 (7.5%) |  |
| IV thrombolysis | 7 (17.5%) | 3 (7.5%) | 0.311 |
| Procedural factors |  |  |  |
| First line technique |  |  | 0.155 |
| - Stent retriever | 14 (35%) | 7 (17.5%) |  |
| - Direct aspiration | 14 (35%) | 21 (52.5%) |  |
| - Combined technique | 12 (30%) | 12 (30%) |  |
| Number of passes | 1 (1, 2) | 2 (1.75, 4) | 0.582 |
| Successful recanalization (mTICI IIb/III) | 38 (95%) | 26 (65%) | 0.002 |
| First pass effect | 20 (50%) | 7 (17.5%) | 0.005 |

DVT, deep vein thrombosis; PTE, pulmonary thromboembolism; Hb, hemoglobin; HbA1c, hemoglobin A1c; LDL, low-density lipoprotein; hsCRP, highly sensitive C-reactive protein; TICI, thrombolysis in cerebral infarction

**Supplemental Data 2.** Distribution of hemorrhagic transformation classification after endovascular thrombectomy


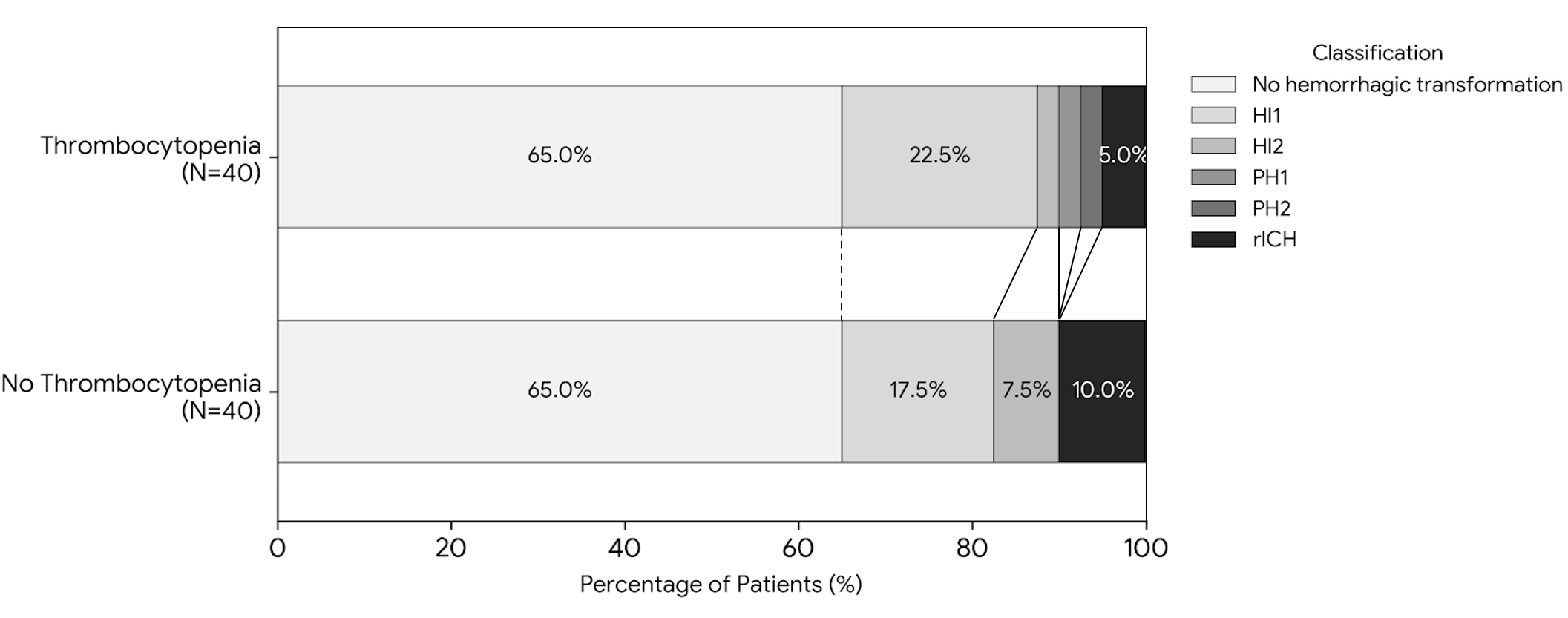


**Supplemental Data 3**. Association analysis of safety and clinical outcomes according to thrombocytopenia status and platelet count tertiles.

| **Any hemorrhagic transformation** | Yes (N=28) | No (N=52) | | *Crude OR* | *p* | *Adjusted OR* | *p* |
| --- | --- | --- | --- | --- | --- | --- | --- |
| Without thrombocytopenia (Reference) | 14 (50%) | 26 (50%) | | Reference |  | Reference |  |
| Thrombocytopenic patients stratified by tertiles of platelet count | | | |  |  |  |  |
| I tertile | 4 (14.29%) | 9 (17.31%) | | 0.825 (0.215-3.168) | 0.780 | 0.544 (0.129-2.296) | 0.407 |
| II tertile | 4 (14.29%) | 9 (17.31%) | | 0.825 (0.215-3.168) | 0.780 | 0.544 (0.129-2.296) | 0.407 |
| III tertile | 6 (21.43%) | 8 (15.38%) | | 1.393 (0.402-4.823) | 0.601 | 1.122 (0.306-4.123) | 0.862 |
| **Symptomatic ICH** | Yes (N=9) | | No (N=71) | *Crude OR* | *p* | *Adjusted OR* | *p* |
| Without thrombocytopenia (Reference) | 4 (44.44%) | 36 (50.70%) | | Reference |  | Reference |  |
| Thrombocytopenic patients stratified by tertiles of platelet count | | | |  |  |  |  |
| I tertile | 2 (22.22%) | 11 (15.49%) | | 1.636 (0.263-10.167) | 0.597 | 2.723 (0.362-20.495) | 0.331 |
| II tertile | 1 (11.11%) | 12 (16.90%) | | 0.750 (0.076-7.381) | 0.805 | 0.997 (0.093-10.665) | 0.998 |
| III tertile | 2 (22.22%) | 12 (16.90%) | | 1.500 (0.243-9.246) | 0.662 | 1.757 (0.272-11.346) | 0.554 |
| **3-month mortality** | Yes (N=37) | No (N=43) | | *Crude OR* | *p* | *Adjusted OR* | *p* |
| Without thrombocytopenia (Reference) | 15 (40.54%) | 25 (58.14%) | | Reference |  | Reference |  |
| Thrombocytopenic patients stratified by tertiles of platelet count | |  | |  |  |  |  |
| I tertile | 8 (21.62%) | 5 (11.63%) | | 2.667 (0.736-9.665) | 0.136 | 3.058 (0.572-16.342) | 0.191 |
| II tertile | 6 (16.22%) | 7 (16.28%) | | 1.429 (0.403-5.059) | 0.580 | 1.256 (0.272-5.790) | 0.770 |
| III tertile | 8 (21.62%) | 6 (13.95%) | | 2.222 (0.645-7.656) | 0.206 | 0.480 (0.076-3.047) | 0.436 |
| **3-month favorable outcomes** | Yes (N=22) | No (N=58) | | *Crude OR* | *p* | *Adjusted OR* | *p* |
| Without thrombocytopenia (Reference) | 15 (68.18%) | 25 (43.10%) | | Reference |  | Reference |  |
| Thrombocytopenic patients stratified by tertiles of platelet count | |  | |  |  |  |  |
| I tertile | 2 (9.09%) | 11 (18.97%) | | 0.303 (0.059-1.558) | 0.153 | 0.497 (0.063-3.923) | 0.507 |
| II tertile | 3 (13.64%) | 10 (17.24%) | | 0.500 (0.118-2.111) | 0.346 | 0.380 (0.057-2.521) | 0.316 |
| III tertile | 2 (9.09%) | 12 (20.69%) | | 0.278 (0.055-1.415) | 0.123 | 0.465 (0.060-3.635) | 0.466 |

OR, odds ratio; mTICI, modified Thrombolysis In Cerebral Infarction; HT, hemorrhagic transformation; ICH, Intracranial hemorrhage; mRS,

Covariates included in the adjusted Firth penalized logistic regression model for hemorrhagic transformation were thrombocytopenia, first line technique, and first pass effect.

Covariates included in the adjusted Firth penalized logistic regression model for sICH were thrombocytopenia, HbA1c, and LDL.

Covariates included in the adjusted Firth penalized logistic regression model for mortality were thrombocytopenia, hemoglobin, D-dimer, systemic metastasis, diabetes and first pass effect.

Covariates included in the adjusted Firth penalized logistic regression model for favorable outcomes were thrombocytopenia, initial NIHSS, hemoglobin, D-dimer, systemic metastasis and first pass effect.

**Supplemental Data 4.** Distribution of modified Rankin Scale (mRS) at 3 months


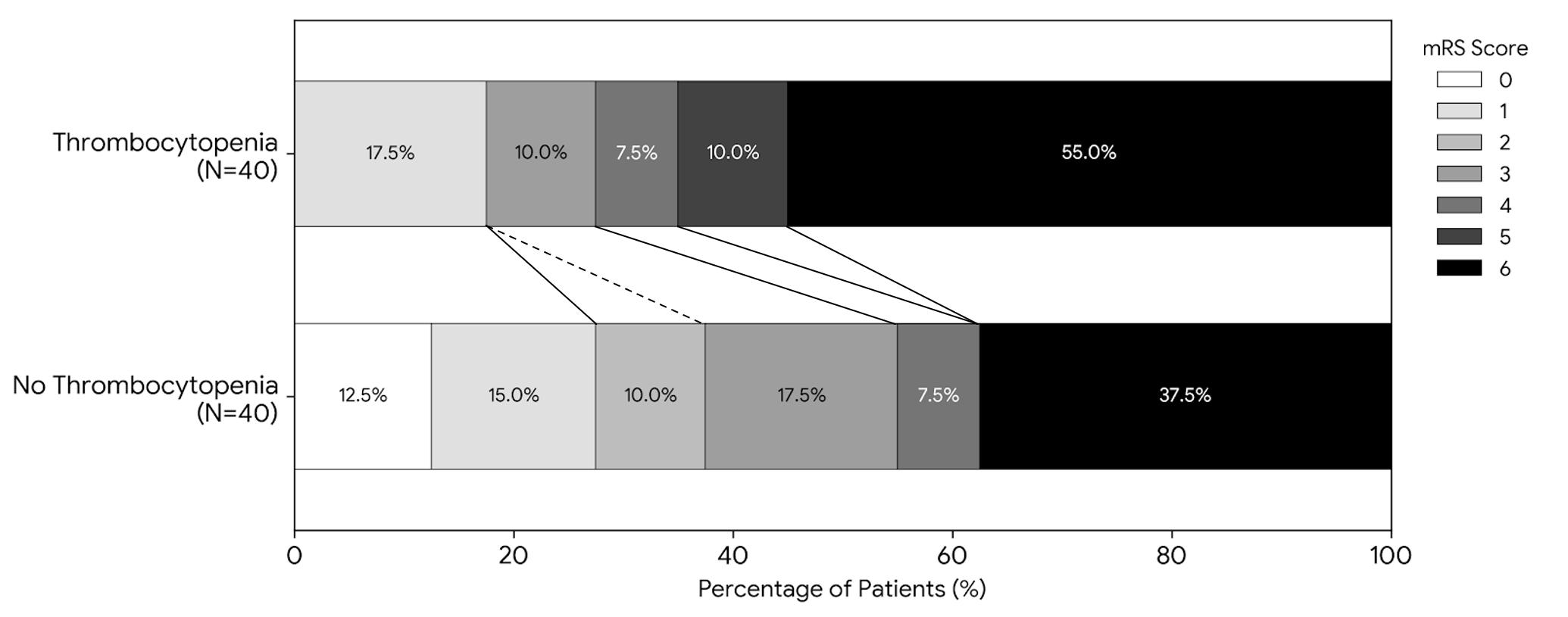


**Supplemental Data 5**. Clinical outcomes stratified by suspected etiologies of thrombocytopenia

|  | Thrombocytopenia without metastasis  (N=17) | Thrombocytopenia with metastasis  (N=23) | *p* |
| --- | --- | --- | --- |
| Hemorrhagic complication |  |  |  |
| Any HT | 6 (35.29%) | 8 (34.78%) | >0.99 |
| Symptomatic ICH | 1 (5.88%) | 4 (17.39%) | 0.546 |
| Clinical outcomes |  |  |  |
| Median mRS score at 3 months (IQR) | 4 (1, 6) | 6 (5, 6) | **0.028** |
| 3-month death | 6 (35.29%) | 16 (69.57%) | 0.067 |
| 3-month favorable outcomes | 5 (29.41%) | 2 (8.7%) | 0.199 |
|  | Thrombocytopenia without chemotherapy  (within 4wks) (N=6) | Thrombocytopenia with chemotherapy  (within 4wks) (N=34) | *p* |
| Hemorrhagic complication |  |  |  |
| Any HT | 0 (0%) | 14 (41.18%) | 0.137 |
| Symptomatic ICH | 0 (0%) | 5 (14.71%) | 0.738 |
| Clinical outcomes |  |  |  |
| Median mRS score at 3 months (IQR) | 4.5 (3, 6) | 6 (4, 6) | 0.647 |
| 3-month death | 3 (50%) | 19 (55.88%) | >0.99 |
| 3-month favorable outcomes | 1 (16.67%) | 6 (17.65%) | >0.99 |
|  | Thrombocytopenia without hypercoagulability  (N=15) | Thrombocytopenia with hypercoagulability  (N=25) | *p* |
| Hemorrhagic complication |  |  |  |
| Any HT | 6 (40%) | 8 (32%) | 0.864 |
| Symptomatic ICH | 1 (6.67%) | 4 (16%) | 0.711 |
| Clinical outcomes |  |  |  |
| Median mRS score at 3 months (IQR) | 4 (1, 6) | 6 (5, 6) | **0.046** |
| 3-month death | 6 (40%) | 16 (64%) | 0.251 |
| 3-month favorable outcomes | 5 (33.33%) | 2 (8%) | 0.107 |

HT, hemorrhagic transformation; ICH, intracranial hemorrhage; mRS, modified Rankin scale; IQR, interquartile range
